# The protective effects of ginsenoside Rg1 against hypertension target-organ damage in spontaneously hypertensive rats

**DOI:** 10.1186/1472-6882-12-53

**Published:** 2012-04-25

**Authors:** Hui Chen, Jun Yin, Yanpin Deng, Min Yang, Lingling Xu, Fukang Teng, Defang Li, Yufan Cheng, Sha Liu, Dong Wang, Tingting Zhang, Wanying Wu, Xuan Liu, Shuhong Guan, Baohong Jiang, Dean Guo

**Affiliations:** 1 Shanghai Institute of Materia Medica, Chinese Academy of Sciences, Haike Road #501, Shanghai, 201203, China; 2 Shenyang Pharmaceutical University, Wenhua Road #103, Shenyang, 110016, China; 3 Fudan University Shanghai Cancer Center, Dongan Road #270, Shanghai, 200023, China; 4 School of Medicine, Shanghai Jiaotong University, Dongfang Road #1630, Shanghai, 200127, China

**Keywords:** Hypertension, Hypertensive complication, Ginsenoside Rg1, Vascular remodeling

## Abstract

**Background:**

Although a number of medicines are available for the management of hypertension, the organ damage induced by hypertension is not resolved. The aim of this study was to investigate the protection of ginsenoside Rg1 (Rg1) against vascular remodeling and organ damage in spontaneously hypertensive rats (SHR).

**Methods:**

Male SHR were treated with 5, 10 or 20 mg/kg Rg1 through intraperitoneal injection per day for 1 month. SHR or Wistar-Kyoto rats (WKY) receiving vehicle (saline) was used as control. Blood pressure detection and pathological stain, transmission electron microscope, immunohistochemical assay were used to elucidate the protection of Rg1.

**Results:**

Blood pressures were not different between control SHR rats and Rg1 treated SHR rats, but Rg1 improved the aortic outward remodeling by lowering the lumen diameter and reducing the media thickness according the histopathological and ultrastructural detections. Rg1 also protected the retinal vessels against inward remodeling detected by immunohistochemical assay. Furthermore, Rg1 attenuated the target heart and kidney damage with improvement on cardiac and glomerular structure.

**Conclusions:**

These results suggested that Rg1 held beneficial effects on vascular structure and further protected against the organ-damage induced by hypertension. These findings also paved a novel and promising approach to the treatment of hypertensive complications.

## Background

Recent evidence suggests that the average blood pressure (BP) in children and adults is rising in the last two decades [1]. Hypertension is becoming one of the main risk factor for cardiovascular and renal vascular disease, and also for mortality around the world [2]. Although the blockers of calcium channel and inhibitors of rennin-angiotensin system are widely applied for clinical therapy, the target-organ damage accompanied by hypertension is still not solved. Therefore, new therapeutic strategies and new medicines to attenuate hypertension-complications are urgently needed [3].

*Panax notoginseng,* one of the most frequently used traditional Chinese medicine, is well known for its efficacy in promoting blood circulation, ameliorating pathological hemostasis, alleviating pain [4-7]. The main active components of *Panax notoginseng* include more than 30 different types of saponins, among which Rg1and Rb1 are found in the highest content. A number of clinical and physiological effects of Rg1 have been described recently, such as inhibition of tubular epithelial to myofibroblast transition [8], improvement of myocardial dysfunction in rats with burn injuries [9], amelioration of hepatic microcirculatory disturbances [10], anti-hyperglycemic activity [11], and improvement of endothelial cell function [12]. Recently, Rg1 has been identified to be an angiogenic factor, which can induce neovascularizaton in vivo and promote proliferation and tubulogenesis of endothelial cells in vitro [13-15]. Further mechanism research revealed that Rg1 could activate phosphatidylinositol-3 kinase Akt pathway, inhibit P38 MARK pathway [16]. Recent studies have demonstrated the beneficial effects of Rg1 on improvement of cardial and renal function [17].

Thus, we carried out the present study in SHR rats to test our hypothesis that Rg1 may inhibit the vascular remodeling and targeted-organ damage induced by hypertension.

## Methods

### Animals and Rg1 treatment

2 month old male Wistar-Kyoto rats (WKY, 280–300 g) and spontaneous hypertension rats (SHR, 280–300 g) were purchased from Shanghai Center of Experimental Animals, Chinese Academy of Sciences. Rats were acclimatized in temperature and humidity-controlled rooms with a 12-h dark/light cycle throughout the study. After 8 week high salt diet, WKY rats that treated by saline were used as normal control (WKY, n = 10). SHR rats were randomly divided into four groups (10 per group): rats that treated by saline were used as hypertension model (SHR); rats in the other three groups were treated by 5 mg/kg Rg1 (SHR-Rg1(5)), 10 mg/kg Rg1 (SHR-Rg1(10)) or 20 mg/kg Rg1 (SHR-Rg1(20)). Saline or Rg 1 (dissolved in saline) were given once a day intraperitoneally. The purity of Rg1 that purchased from Shanghai Yousi Bio-Tech Co., Ltd. was more than 99% evaluated by high-performance liquid chromatography ( Additional file 1: Figure S1) and the chemical structure of Rg1 was elucidated by ^13^ C NMR ( Additional file 2: Figure S2Additional file 3: Figure S3), which is in agreement with those previous report [18]. 8.0% high salt diet was fed during the whole research, and saline or Rg1 was given from the ninth week of experiment for 4 weeks. The whole experiment protocol was shown in Additional file 4 Figure S4. Experimental procedures were approved by the institute animal ethics committee (SIMM-AE-GDA-2010-05) and were in accordance with the National Institute of Health guidelines.

### Measurement of blood pressure in conscious rats

Systolic blood pressure (SBP) and diastolic pressure (DBP) were measured 0.5 h after the administration of Rg1 at the indicated time ( Additional file 4: Figure S4) using the tail-cuff method. Briefly, the rats were placed in a restrainer with heating pad. The blood pressure was continuously recorded by a tail-cuff apparatus (ALC-NIBP, Shanghai Alcott Biotech Co., China) that was controlled with a computer after stabilizing at 37°C for at least 10 min.

### Measurements of hemodynamic parameters

The rats were anesthetized, and a Mikro-tipped SPR-320 catheter (Millar Instruments Inc) was inserted through the right carotid artery into left ventricle. Heart rate, mean arterial pressure (MAP), left ventricular systolic pressure (LVSP), end-diastolic pressure (EDP) of rats were recorded by PowerLab 8/30 instrument (ADInstruments, Australia). Maximal rate of pressure development for contraction (+dP/dt_max_) and maximal rate of pressure development for relaxation (−dP/dt_max_) were all calculated from the continuously collected pressure signal.

### Histopathological detection

After the treatment of Rg1, ascending aorta, heart and kidney of each rat were weighed, and then fixed by 4% neutral-buffered paraformaldehyde for 24 h. Heart index was calculated as that the heart weight was divided by body weight. All the specimens were paraffin-embedded, cut at 5 μm and were stained with haematoxylin and eosin. Photomicrographs were taken using an Olympus BX51 microscope plus Olympus DP71 CCD camera (Olympus Corporation, Japan). Software Image-Pro Plus version 6.0 was used to detect lumen diameter and media thickness of aorta. The aortic lumen diameter was calculated as the mean of the inner diameters through the center of vascular circle, and the media thickness defined as the distance between the internal and external elastic lamina. At least 16 values were measured from the points distributed evenly on every aorta. Paraffin-embedded slices were also stained with 0.1% picric sirius red (Sigma-Aldrich Inc, St Louis, USA) for fibrosis detection.

### Immunohistochemical detection on retinal vessels

Rat eyes were fixed in 4% neutral-buffered paraformaldehyde and the solution was replaced every two days. Before retinal dissection, the eyes were washed by running water for 20 mins. Retinas were dissected, flattened on slides, washed by PBS, then incubated in PBS containing 0.5% Triton X-100 and 10% normal goat serum for 1 h at room temperature. After a short rinse with PBS, retinas were incubated with the α-SMA-Cy3 (Sigma-Aldrich, Germany) and lectin-FITC (Sigma-Aldrich, Germany) at 1000 times dilution at 37°C for 1 h. Retinal artery could be stained by both α-SMA-Cy3 and lectin-FITC positively. Photomicrographs were taken with Olympus BX51 fluorescence microscope and retinal vessel diameter was directly measured at the point of 0.5 mm from the middle of the arteriole.

### Transmission electron microscopy

Aorta, heart, kidney samples were dissected, cut into small pieces and fixed with 2.5% glutaraldehyde in 0.1 M sodium cacodylate buffer for 2 h in 4°C. The specimens were then rinsed, post-fixed in cacodylate -buffered 2% osmium tetroxide, and embedded as monolayers in LX-112 (Ladd Research Industries, USA). Ultrathin sections were contrasted with uranyl acetate followed by lead citrate and observed with a Tecnai 12 BioTwin transmission electron microscope (Philips Electronic Instruments, USA). Random sections were selected for analysis by an electron microscopy technician blinded to the treatments.

### Data analysis

All quantitative values are given as mean ± S.E. Mean values of data from different treatment groups were compared using one-way ANOVA. After confirming the equal variances, unpaired Student’s *t*-test was used to compare difference between means of two groups using SigmaPlot software. *P* < 0.05 was considered to be statistically significant.

## Results

### No influence of Rg1 on blood pressure

**Figure 1:**
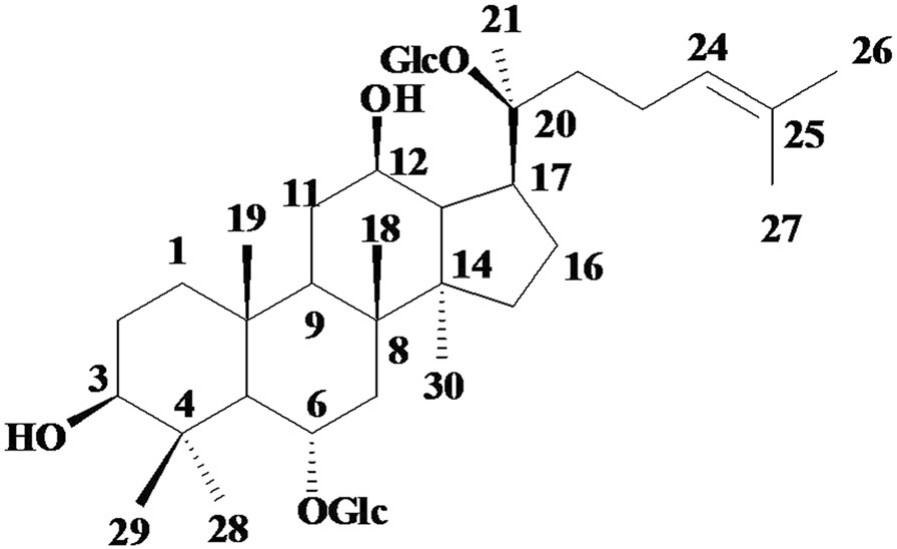
Chemical structure of Rg1.

The structure of Rg1 was shown in Figure 1. Hypertension is well established as a stimulus for cardiovascular remodeling. It was therefore important to determine whether Rg1 regulated blood pressure or not. Throughout the experiments, the blood pressure in the four SHR groups were significantly higher than in the WKY group, but no regulation was found for Rg1 on blood pressure during the whole experiment using tail-cuff method. Furthermore, Rg1 did not show considerable regulation on left ventricular function demonstrated by HR, MAP, +dP/dt_max_, -dP/dt_max_ and LVSP detected by Millar catheter (Table 1).

**Table 1:** Effects of Rg1 on blood pressure

	**WKY**	**SHR**	**SHR-Rg1(5)**	**SHR-Rg1(10)**	**SHR-Rg1(20)**
SBP(mmHg)	133.7 ± 2.2	184.5 ± 3.4^###^	186.0 ± 2.4^###^	183.5 ± 3.3^###^	183.0 ± 4.2^###^
DBP(mmHg)	97.5 ± 2.0	139.0 ± 3.0^###^	138.0 ± 2.4^###^	139.1 ± 2.8^###^	133.8 ± 3.9^###^
HR (bpm)	360.6 ± 14.0	393.6 ± 12.5^#^	366.7 ± 13.7	412.6 ± 8.9	433.5 ± 9.3*
MAP(mmHg)	111.5 ± 6.3	170.1 ± 16.7^###^	181.6 ± 14.0^###^	175.4 ± 37.4^###^	193.7 ± 10.4^###^
EDP(mmHg)	6.1 ± 1.7	7.5 ± 3.0	3.1 ± 1.0	4.8 ± 3.9	5.5 ± 1.8
LVSP(mmHg)	120.9 ± 7.1	207.1 ± 17.9^###^	213.7 ± 21.1^###^	224.3 ± 35.4^###^	217.5 ± 16.8^###^
+dP/dt_max_ (mm HgS^-1^)	8661.1 ± 717.7	11735.7 ± 635.8^##^	13063.6 ± 470.8^###^	12651.9 ± 803.0^##^	12636.7 ± 514.7^###^
-dP/dt_max_ (mm HgS^-1^)	−7514.6 ± 626.0	−11272.1 ± 872.8^##^	−12410.0 ± 595.7^###^	−11330.2 ± 722.8^###^	−11464.1 ± 534.8^###^
HW/BW(mg/g)	2.9 ± 0.2	3.9 ± 0.05^#^	3.8 ± 0.08^#^	3.8 ± 0.07^#^	3.6 ± 0.06^# **^

All the values are expressed as mean ± S.E. Systolic pressure (SBP), diastolic pressure (DBP), heart rate (HR), mean arterial pressure (MAP), maximum rate of pressure development for relaxation (−dP/dt_max_), maximum rate of pressure development for contraction (+dP/dt_max_), left ventricular systolic pressure (LVSP) were demonstrated. Heart weight divided by body weight (HW/BW) is also calculated. MAP, EDP, LVSP, +dp/dt_max_, -dp/dt_max_ were acquired using Millar catheter and recorded by PowerLab 8/30 instrument (ADInstruments, Australia). Heart rate, SBP and DBP were acquired by tail-cuff apparatus. Mean values of data from different treatment groups were compared using one-way ANOVA. After confirming the equal variances, unpaired Student’s *t*-test was used to compare difference between means of two groups using SigmaPlot software. ^#^*P* < 0.05, ^###^*P* < 0.001 versus WKY; **P < 0.01, ***P < 0.001 versus SHR. n = 10 for each group.

### Rg1 reduces aortic remodeling

**Figure 2:**
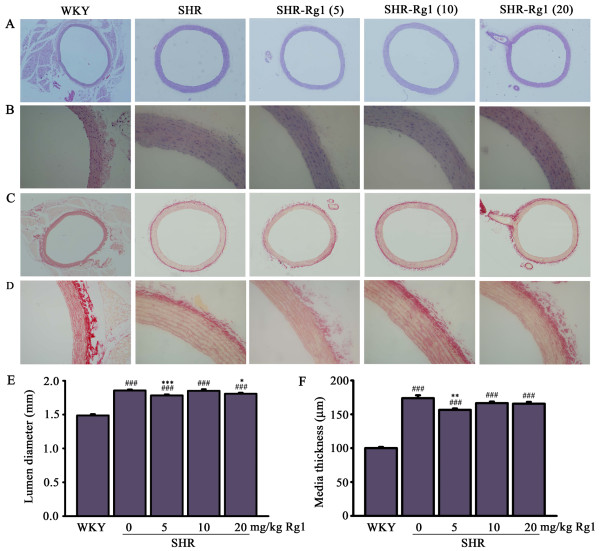
**Effects of Rg1 on aorta structure. (A)** Representative sections of the aortas staining with hematoxylin-eosin (magnification 40X). **(B)** Representative sections of the aortas staining with hematoxylin-eosin (magnification 200X) **(C)** Representative sections of the aortas staining with Sirius red ((magnification 40X) **(D)** Representative sections of the aortas staining with Sirius red (magnification 200X) **(E)** Quantitative data of lumen diameter. **(F)** Quantitative data of media thickness. ###*p* < 0.001 compared with WKY, ***p < 0.001 compared with SHR. n = 4 for each group.

Aortic structure was investigated by histopathological analysis using haematoxylin and eosin stain and shown in Figure 2A and Figure 2B. The cell alignment was un-regular and cell density was reduced in SHR relative to WKY, and Rg1 treatment markedly attenuated this phenomenon (Figure 2B). Vascular fibrosis that impairs vascular contractility was detected by picro sirus stain. No significant improvement of Rg1 on vascular fibrosis was found (Figure 2C, D). It is well known that long term hypertension could induce vascular remodeling including greater values of lumen diameter and media thickness. The lumen diameter was 1.86 ± 0.02 mm in SHR rats compared with 1.49 ± 0.02 mm in WKY rats (*P* < 0.001). Rg1 decreased this outward remodeling significantly (*P* < 0.001 for 5 mg/kg Rg1; *P* < 0.05 for 20 mg/kg Rg1; Figure 2E). Furthermore, the thickening of aortic media was clearly observed in the arteries of SHR comparing with WKY (173.7 ± 4.3 μm versus 100.0 ± 1.7 μm; *P* < 0.001), and the media thickness in 5 mg/kg Rg1 treated SHR decreased to 156.6 ± 2.1 μm (*P* < 0.01; Figure 2F).

### Rg1 reduces retinal vascular remodeling

**Figure 3:**
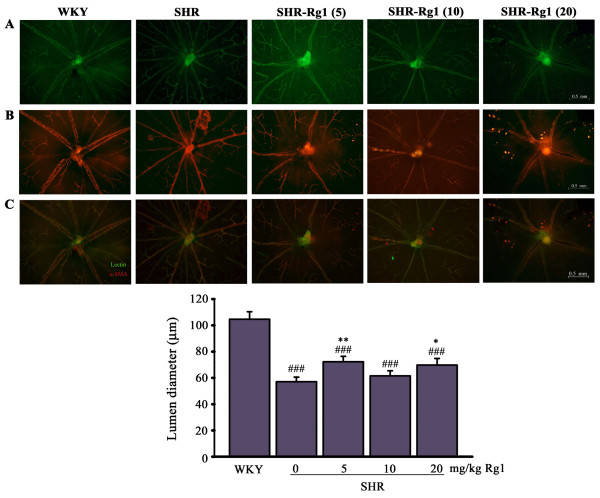
**Effects of Rg1 on retinal remodeling induced by hypertension.** Whole-mount double-staining of retinal vessels with FITC-coupled lectin (green) and α-SMA antibody (red). **(A)** lectin positive arteries. **(B)** α-SMA–positive arteries. **(C)** The merged picture for both **(A)** and **(B)**. **(D)** Quantitative data of lumen diameter for retinal arteries. ###*p* < 0.001 compared with WKY; **p < 0.01, **p < 0.01 compared with SHR. n = 4 for each group.

Hypertension is a strong stimulus not only on conductance vessel such as aorta, but also on resistance vessel such as retinal arteries [19]. In the present study, we investigated effects of Rg1 on the remodeling of small retinal arteries (Figure 3). Arteries and veins were distinguished by double-staining of the retinal blood vessels with lectin-FITC and an anti-α-SMA-Cy3 antibody, which resulted in a more pronounced α-SMA staining of small arteries and arterioles. The remodeling of retinal arteries was evaluated by arteries diameter at the point 0.5 mm to the middle of the arteriole. Hypertension induced considerable decrease on retinal arterial diameter in SHR (57.06 ± 3.47 μm) compared with WKY (104.65 ± 5.695 μm; *P* < 0.001). While Rg1 inhibited this inward remodeling induced by hypertension, the diameter values were 72.2 ± 4.2 μm (SHR-Rg1(5)), 61.5 ± 3.9 μm (SHR-Rg1(10)) and 69.8 ± 5.0 μm (SHR-Rg1(20)), respectively. 

### Rg1 protects the ultra-structural integrity of aorta

**Figure 4:**
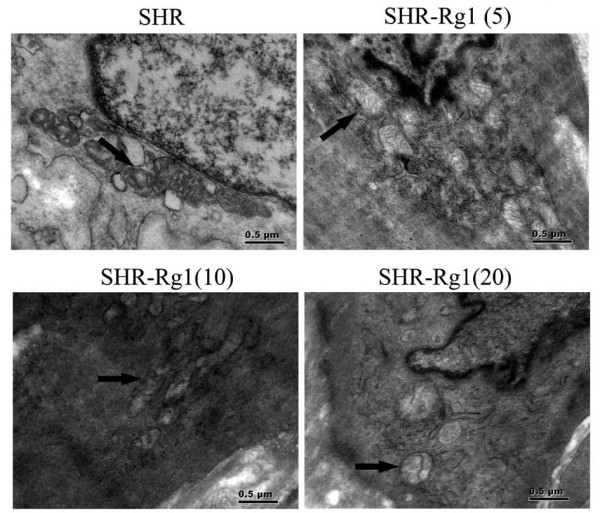
**Representative photographs of transmission electron microscopy for vascular smooth muscle cells.** Arrows indicate mitochondria. n = 3 for each group. The representative figures were shown.

The ultra**-**structure of aorta was examined using transmission electron microscopy (Figure 4). Vascular smooth muscle cell is the main cell type of aorta and play important role on vascular function. In the present study, we detected the mitochondrial ultra-structure of smooth muscle cells of aorta. The mitochondria of SHR showed irregularly and spherical shaped, especially swollen with electron-lucent matrix. With Rg1 treatment, although the matrix of mitochondria was partial disruption, but no-swollen cristae was detected.

### Rg1 protects against the impairment of hypertensive heart and kidney

**Figure 5:**
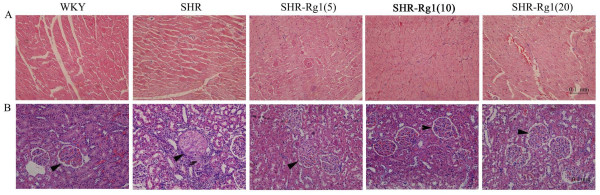
**Rg1 improved heart and kidney structure of SHR detected by hematoxylin-eosin stain. (A)** Representative photomicrographs from heart tissue of WKY, SHR, SHR-Rg1(5), SHR-Rg1(10), SHR-Rg1(20) with 200 X magnification. (**B**) Representative photomicrographs from kidney tissue of WKY, SHR, SHR-Rg1(5), SHR-Rg1(10), SHR-Rg1(20) with 200 X magnification. n = 4 for each group.

Cardial and renal deterioration are both complications in patients with hypertension. Histopathological detection was performed in order to investigate the protective effects of Rg1 on structure of heart and kidney in SHR. Cardiomyocyte hypertrophy was a significant characteristic in SHR compared with WKY, and this impairment was attenuated in SHR-Rg1 groups (Figure 5A). This finding is consistence with the result of heart index, the values were 2.9 ± 0.2 mg/kg (WKY), 3.9 ± 0.05 mg/kg (SHR), 3.8 ± 0.08 mg/kg (SHR-Rg1(5)), 3.8 ± 0.07 mg/kg (SHR-Rg1(10)), 3.6 ± 0.06 mg/kg (SHR-Rg1(20)), respectively (Table 1). The typical photomicrographs of glomeruli were shown in Figure 5B. Glomeruli of SHR showed prominent hyalinization (mesangial thickening) comparing with WKY rats. SHR treated with Rg1 showed the reduction in the development of hyalinization.

### Rg1 protects against cardio-fibrosis induced by hypertension

**Figure 6:**
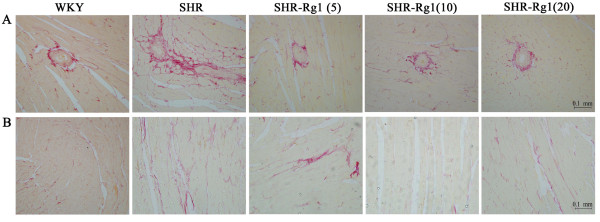
**Rg1 decreased heart fibrosis induced by hypertension. (A)** Representative perivascular area of whole heart stained by Sirius red. (**B**) Representative few-vascular area of whole heart stained by Sirius red. The position of collagen deposition was stained in red. n = 4 for each group.

Myocardial fibrosis, the common end point of hypertensive heart disease, has been linked to the development of contractile dysfunction and cardiac failure. Picro sirus stain showed the fibrotic area was larger in SHR compared with WKY at the perivascular area. With Rg1 treatment, the fibrotic areas were considerably smaller than SHR (Figure 6A). Thicker and darker stain of collagen fiber was seen on SHR compared with WKY at the few-vascular area; while collagen fiber became thinner, weaker and more discontinuous with Rg1 treatment (Figure 6B). No significant improvement of Rg1 on renal fibrosis was detected (data not shown).

### Rg1 protects the ultra-structure integrity of heart and kidney of SHR

**Figure 7:**
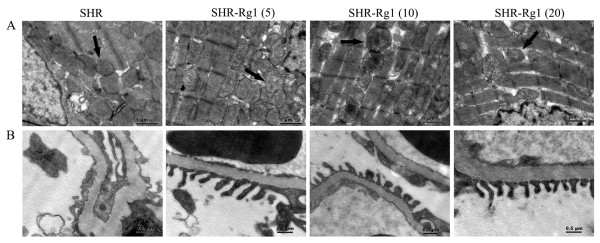
**Rg1 improved ultra-structure of heart and kidney. (A)** Electron micrograph of mitochondria marked by arrow in heart. **(B)** Electron micrograph of glomerular basement membrane. n = 3 for each group. The representative figures were shown.

The damage on mitochondria of cardiomyocyte included swelling, disorganization or even disappearance of cristae in SHR group. While, no overt ultra-structural abnormality was observed in ventricular samples from Rg1 treated groups, with relative organized mitochondria (Figure 7A). Electron microscopy also demonstrated a heterogeneous thickening of the glomerular basement membrane, accompanying irregular arrangement or inter-adhesion of the podocytes in SHR group. With Rg1 treatment, podocytes were in very regular array (Figure 7B).

## Discussion

This study assessed the protective effects of Rg1 on hypertension target-organ damage in SHRs. The main findings are as follows: (i) Rg1 attenuated the outward remodeling of aorta. (ii) Rg1 decreased the inward remodeling of small artery. (iii) Rg1 improved the cardial hypertrophy and fibrosis. (iv) Rg1 maintained the normal structure of kidney.

Numerous studies have demonstrated that high salt intake causes adverse structural and functional effects in the cardiovascular system [20,21]. Excessive salt intake is often associated with an increase in arterial pressure and, consequently, increases in arterial pressure may partially mediate salt-related adverse effects [22]. In addition to the well-admitted effect of sodium on blood pressure, several clinical and experimental observations are in favour of non-pressure-related effects of salt that could contribute to its influence on cardiovascular outcome [23]. High salt intake caused hypertrophic response, then concentric cardiac-remodeling [24]. High dietary salt led to widespread fibrosis and increased TGF-β1 in the heart and kidney in normotensive and hypertensive rats, suggesting that excessive salt intake may be an important direct pathogenic factor for cardiovascular disease [21].

Up to now, cardiovascular disease is still the most important factor that affects people’s life, especially the hypertension [1,2,25]. To delay or prevent hypertension target-organ, containing heart failure and renal failure, is essential to improve patient’s quality of life [26]. In agreement with previous reports that the most common change found in large arteries in hypertension is an “outward hypertrophic remodeling”, we detected the increased lumen diameter and wall thickness in SHR compared with WKY. In hypertensive large arteries, these changes appear to be the consequence of cellular hypertrophy, cell hyperplasia, increased deposition of fibrillar or nonfibrillar matrix, or from a combination of these events [27-30]. Remodeling of the small resistance arteries may involve an inward or an outward remodeling [19,31]. In the present study, we confirmed an inward remodeling in small retinal arteries of SHR compared with WKY. Rg1 treatment not only reduced the outward remodeling of large conductance arteries but also attenuated the inward remodeling of small resistance arteries, although no regulation of Rg1 on blood pressure was found. These results agree with those of other reports that ACE inhibitors are effective in controlling or reversing vascular remodeling, not depending on the anti-hypertensive effects [32,33].

The final goal of novel therapy of hypertension is not only to normalize the blood pressure level but also to prevent end-organ damage, such as cardiac hypertrophy and renal dysfunction [26,34]. Altered retinal arteries diameter has also been demonstrated to be associated with heart failure, suggesting that evaluation of the retinal microvasculature may be a useful predictor of target organ damage [35,36]. Collagen deposition is the risk factor which plays an important role in development of organ failure, such as heart failure and renal failure [37]. The myocardial matrix becomes less distensible, as the formation of the adducts in collagen resists normal turnover. Therefore, monitoring cardiac fibrosis and use of medicines that reverse collagen accumulation might represent a novel opportunity to alter the natural history of hypertensive heart disease.

In the previous report, the presence of myocardial hypertrophy and fibrosis in SHR was obvious at early stage, but the diastolic and systolic dysfunction did not occur until 13 to 18 months of age of SHR [38]. This finding is in consistence with our present study. It is hopeful that the improvement of Rg1 on cardiac function could be detected by elongation of Rg1 treatment or selection of SHR more than 13 month old, basing on the anti-fibrotic effects of Rg1 at the early stage of hypertension.

We detected heart rate on conscious SHR during the experiment using tail-cuff apparatus, and up-regulation of Rg1 on heart rate was found. Increase of heart rate induced by Rg1 should be considered as a side effect in cardiovascular disease. Shen et al. have reported that Rg1 down-regulated heart rate in anesthetized mice treated by glutamate [39]. The difference between our report and Shen et al. maybe derive from the different species used, suggesting the increase of Rg1 on heart rate may not occur if animal model other than SHR was used. Different biological response induced by extract from herbs between species was also reported [40,41]. Further study was necessary to clarify the effects of Rg1 on heart rate on different species and the underlying mechanism.

## Conclusion

In summary, Rg1 performed cardiac and renal protection with inhibition of vascular remodeling not only on large conductance artery but also on small resistance artery. The unique structure and efficiency of Rg1 afford a great deal of potential for further optimization of this natural compound into therapeutics for hypertension related abnormalities.

## Competing interest

The authors declare that they have no competing interests.

## Authors’ contributions

Hui Chen, Yangping Deng, Lingling Xu and Tingting Zhang performed animal experiments. Min Yang, Wanying Wu and Shuhong Guan performed Chemical analysis, Fukang Teng, Defang Li, Yufan Cheng, Sha Liu, Dong Wang, Xuan Liu performed histological and immunohistochemical detection. Jun Yin, Baohong Jiang and De-an Guo designed, analyzed experiments and prepared the manuscript. All authors read and approved the final manuscript.

## Pre-publication history

The pre-publication history for this paper can be accessed here:

http://www.biomedcentral.com/1472-6882/12/53/prepub

## Supplementary Material

Additional file 1 **Figure S1.** The representative chromatogram of high-performance liquid chromatography for Rg1. Agilent 1100 HPLC system was used and the detection wavelength was set at 280 nm. The mobile phase consisted of **(A)** acetonitrile and **(B)** 0.05% aqueous trifluoroacetic acid (V/V), using a gradient elution of 2%-10% A at 0–7 min, 10%–30% A at 7–20 min, 23%–27% A at 20–35 min, and 27%–60% A at 35–50 min . Click here for file

Additional file 2 **Figure S2.**^13^C NMR spectrum of Rg1 detected by Bruker AM-400 spectrometer. Click here for file

Additional file 3 **Figure S3.** Structure elucidation of Rg1. **(A)** Chemical structure of Rg1. **(B)**^13^C NMR (100 MHz) spectral data for Rg1. Click here for file

Additional file 4 **Figure S4.** Experimental protocol. Rg1 treatment was performed from week 9 to week 12. Click here for file
