# Supplementary figures and images for: The protective effects of ginsenoside Rg1 against hypertension target-organ damage in spontaneously hypertensive rats
Source: BMC Complement Altern Med. 2012 Apr 25;12:53. doi: 10.1186/1472-6882-12-53 (PMC3453499; doi:10.1186/1472-6882-12-53)

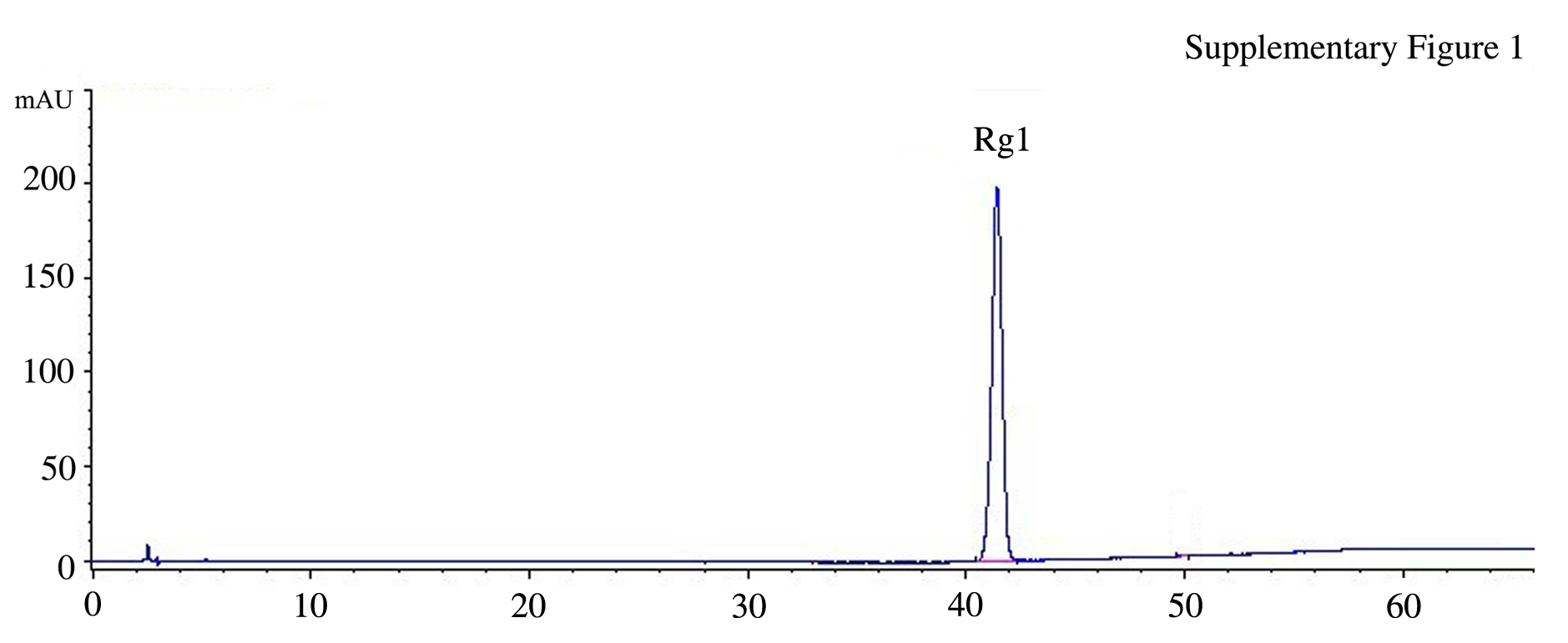

Supplement: Additional file 1 — Figure S1. The representative chromatogram of high-performance liquid chromatography for Rg1. Agilent 1100 HPLC system was used and the detection wavelength was set at 280 nm. The mobile phase consisted of (A) acetonitrile and (B) 0.05% aqueous trifluoroacetic acid (V/V), using a gradient elution of 2%-10% A at 0–7 min, 10%–30% A at 7–20 min, 23%–27% A at 20–35 min, and 27%–60% A at 35–50 min . [file 1472-6882-12-53-S1.tiff]

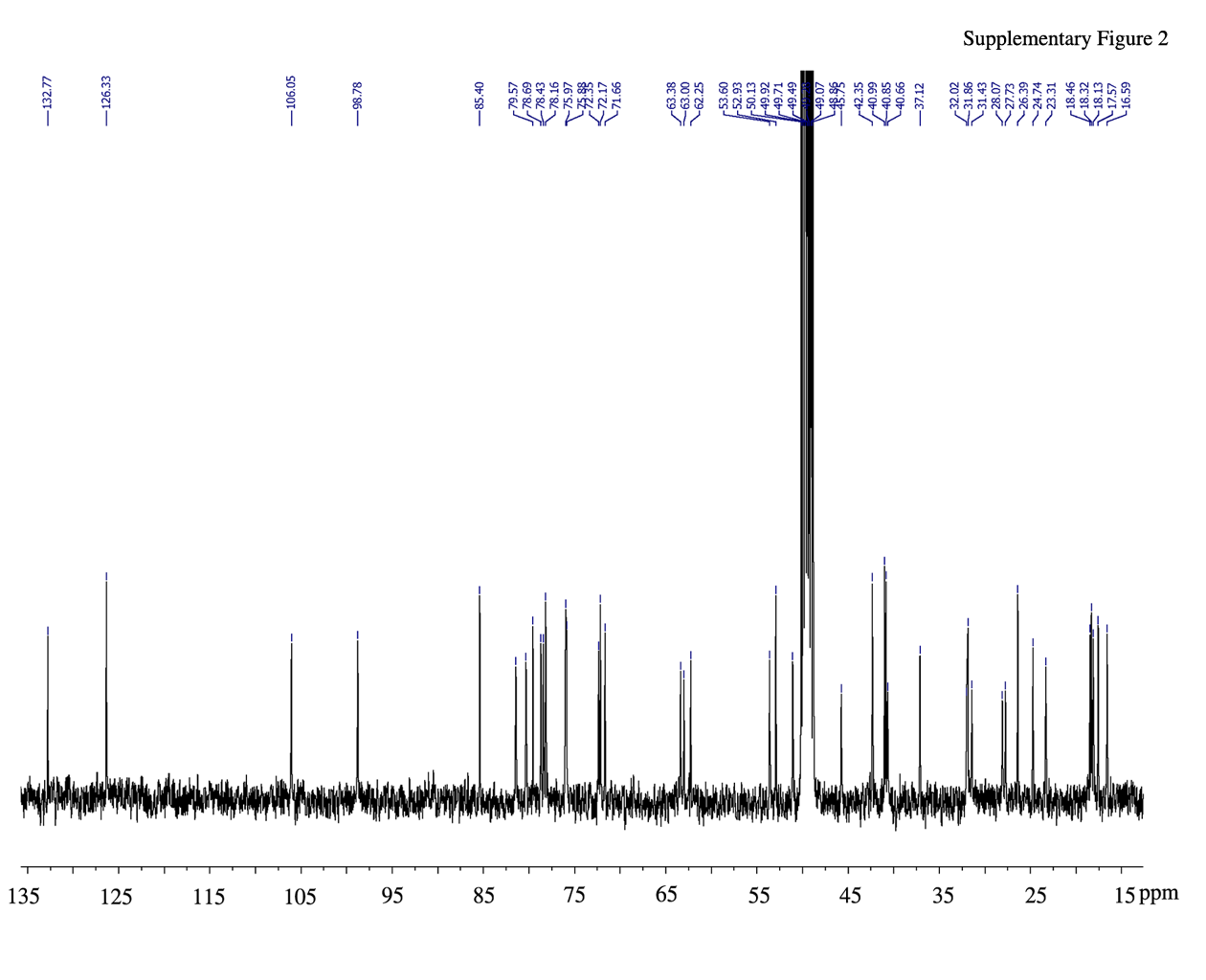

Supplement: Additional file 2 — Figure S2.13C NMR spectrum of Rg1 detected by Bruker AM-400 spectrometer. [file 1472-6882-12-53-S2.tiff]

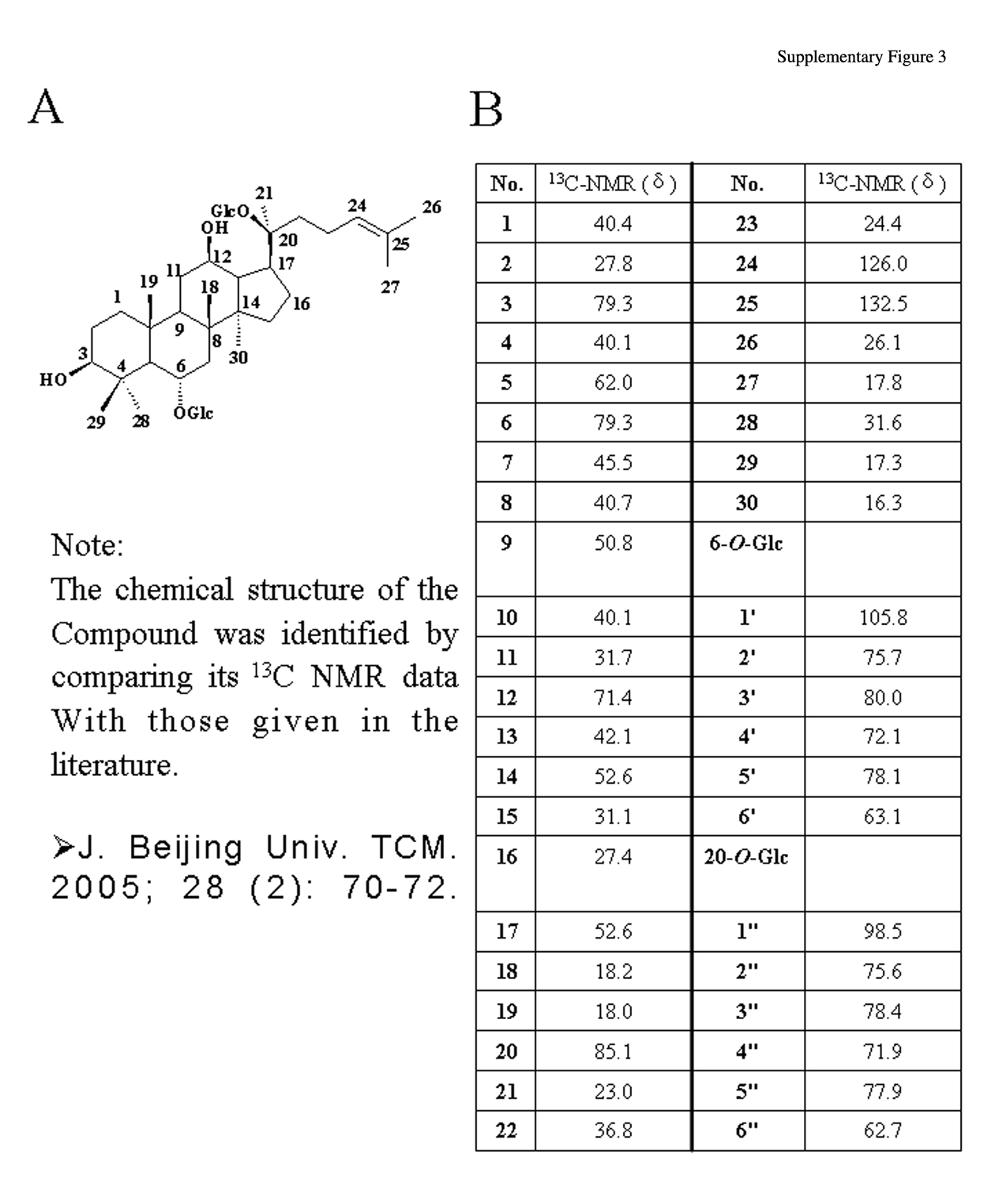

Supplement: Additional file 3 — Figure S3. Structure elucidation of Rg1. (A) Chemical structure of Rg1. (B)13C NMR (100 MHz) spectral data for Rg1. [file 1472-6882-12-53-S3.tiff]

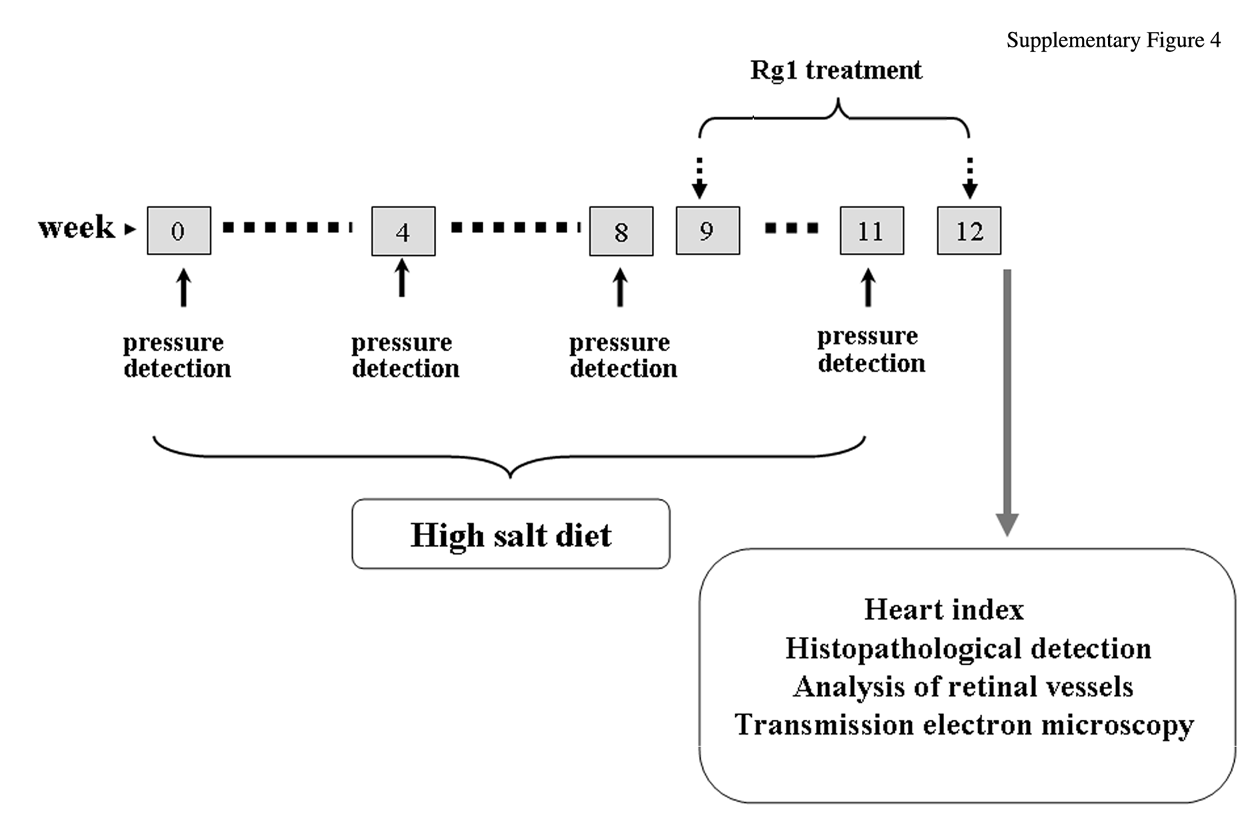

Supplement: Additional file 4 — Figure S4. Experimental protocol. Rg1 treatment was performed from week 9 to week 12. [file 1472-6882-12-53-S4.tiff]
